# Does HTLV-1 Infection Show Phenotypes Found in Sjögren’s Syndrome?

**DOI:** 10.3390/v14010100

**Published:** 2022-01-06

**Authors:** Hideki Nakamura, Masako Tsukamoto, Yosuke Nagasawa, Noboru Kitamura, Toshimasa Shimizu, Atsushi Kawakami, Kinya Nagata, Masami Takei

**Affiliations:** 1 Division of Hematology and Rheumatology, Department of Medicine, Nihon University School of Medicine, Tokyo 173-8610, Japan; tsukamoto.masako@nihon-u.ac.jp (M.T.); nagasawa.yosuke@nihon-u.ac.jp (Y.N.); kitamura.noboru@nihon-u.ac.jp (N.K.); nagata.kinya@nihon-u.ac.jp (K.N.); takei.masami@nihon-u.ac.jp (M.T.); 2 Division of Advanced Preventive Medical Sciences, Department of Immunology and Rheumatology, Graduate School of Biomedical Sciences, Nagasaki University, Nagasaki 852-8501, Japan; toshimasashimizu2000@yahoo.co.jp (T.S.); atsushik@nagasaki-u.ac.jp (A.K.)

**Keywords:** follicular dendritic cell, HTLV-1, salivary gland epithelial cell, Sjögren’s syndrome, viral infection

## Abstract

Viruses are a possible cause for Sjögren’s syndrome (SS) as an environmental factor related to SS onset, which exhibits exocrine gland dysfunction and the emergence of autoantibodies. Although retroviruses may exhibit lymphocytic infiltration into exocrine glands, human T-cell leukemia virus type 1 (HTLV-1) has been postulated to be a causative agent for SS. Transgenic mice with HTLV-1 genes showed sialadenitis resembling SS, but their phenotypic symptoms differed based on the adopted region of HTLV-1 genes. The dominance of *tax* gene differed in labial salivary glands (LSGs) of SS patients with HTLV 1-associated myelopathy (HAM) and adult T-cell leukemia. Although HTLV-1 was transmitted to salivary gland epithelial cells (SGECs) by a biofilm-like structure, no viral synapse formation was observed. After infection to SGECs derived from SS patients, adhesion molecules and migration factors were time-dependently released from infected SGECs. The frequency of the appearance of autoantibodies including anti-Ro/SS-A, La/SS-B antibodies in SS patients complicated with HAM is unknown; the observation of less frequent ectopic germinal center formation in HTLV-1-seropositive SS patients was a breakthrough. In addition, HTLV-1 infected cells inhibited B-lymphocyte activating factor or C-X-C motif chemokine 13 through direct contact with established follicular dendritic cell-like cells. These findings show that HTLV-1 is directly involved in the pathogenesis of SS.

## 1. Introduction

A controversial question is whether retroviruses (including human T-cell leukemia virus [HTLV-1] [1,2,3,4], human immunodeficiency virus [HIV] [5,6,7], Epstein–Barr virus [EBV] [8,9], and hepatitis C virus [HCV] [10,11,12]) and/or cytomegalovirus [13,14] are candidate causes of Sjögren’s syndrome (SS), which is an autoimmune disease exhibiting sialadenitis, the appearance of autoantibodies including anti-Ro/SS-A, La/SS-B antibodies, and extraglandular manifestations [15,16,17]. These reports are mostly from the late 1980s and early 1990s, when the link between viral infections and SS was of particular interest. The detection of some genes and proteins of the retrovirus in the salivary glands of patients with SS instead of the retrovirus itself was also reported at that time [18,19]. In particular, Garry et al. reported that type A retrovirus particles similar to the HIV virus itself were detected in lymphoblast cells in SS salivary gland tissue [18]. Their report provided an opportunity for the development of research about the possibility that HIV and HTLV-1 are directly involved in the onset of SS.

The identification of retrovirus particles and the proof of antibodies against viruses in the sera of SS patients has increased the importance and position of retrovirus infection in the pathogenic mechanism of SS. In addition to retroviruses, EBV and HCV have also been proposed as candidate microorganisms that cause inflammation of salivary glands, but a detailed description of EBV must be left to another article. Haddad et al. investigated the formation of lymphocyte foci in the lip salivary glands in patients with HCV-positive hepatitis or cirrhosis and in uninfected corpses, and their results confirmed obvious salivary gland inflammation in HCV-positive cases; they reported that the inflammation closely resembled salivary gland inflammation in SS [10]. Koike et al. created transgenic mice with the HCV envelope gene, which did not induce hepatitis; however, the mice exhibited exocrine inflammation of the salivary and lacrimal glands [20]. This confirmed that HCV is a virus that can cause SS-like pathology.

Although there are few reports on cytomegalovirus (CMV) and SS compared to the above-mentioned viruses, some important descriptions of an association between CMV and SS have been obtained. Shillitoe et al. reported in 1982 that patients with SS had significantly high IgG and IgM class antibody levels against CMV [21], but an association between CMV and SS was not observed in later serological studies [22,23]. An animal model infected with murine CMV showed sialadenitis [24], but an in situ hybridization study examining CMV genes in labial salivary glands (LSGs) demonstrated no detection of a signal against CMV in LSGs of SS patients [14].

HTLV-1, the central microorganism in this review, is a type of ribonucleic acid (RNA) virus. In addition to the three regions of *gag*, *pol*, and *env* [25,26], which are the basic skeletons of retroviruses, the presence of a *pX* region is a major feature of HTLV-1. It was long thought that the *tax* gene derived from the *pX* region that is present in the plus strand of HTLV-1 plays a central role among the genes that drive HTLV-1, because Tax protein was shown to have the ability to activate nuclear factor kappa B (NF-κB) [27], which is involved in the activation of proinflammatory cytokines. However, the clarification of the existence of HTLV-1 bZIP factor (*HBZ*) [28,29] derived from the minus strand contributed to the subsequent development of virology and clinical virology. For example, Basbous et al. showed in 2003 that HBZ has the potential to negatively regulate Tax activity by reducing the DNA binding ability of c-Jun and JunB [29].

HTLV-1 was isolated by Gallo et al. in 1980 [30], but in 1977 Takatsuki et al. had already recognized the presence of abnormal lymphocytes with notches in adult T-cell leukemia (ATL), a type of hematological malignancy [31]. After Hinuma et al. reported that the causative microorganism of ATL was HTLV-1 based on their isolation of HTLV-1 and detection of antibodies in human sera [32], Yoshida et al. reported the results of a whole-genome analysis of HTLV-1 [33]. In addition, HTLV-1-associated myelopathy (HAM), which exhibits slowly progressive myelopathy, was reported by Osame et al. [34], and HTLV-1-associated uveitis, which appears in HTLV-1 carrier patients, was reported by Mochizuki et al. [35]. Japanese researchers have thus made significant contributions to the elucidation of HTLV-1-related diseases. Although the number of HTLV-1 carriers in Japan has decreased due to measures concerning blood products and breast milk implemented by the Japanese government, it was suggested in 2012 that there are many HTLV-1-infected carriers among elderly Japanese [36]. The existence of individuals who are newly infected with HTLV-1 has been clarified by a report from the Japanese Red Cross Society [37]. On the other hand, SS has not yet been recognized as an HTLV-1-related disease, and this review discusses whether there is a direct association between SS and HTLV-1.

## 2. Animal Models

### 2.1. Tax Transgenic Mice

**Figure 1:**
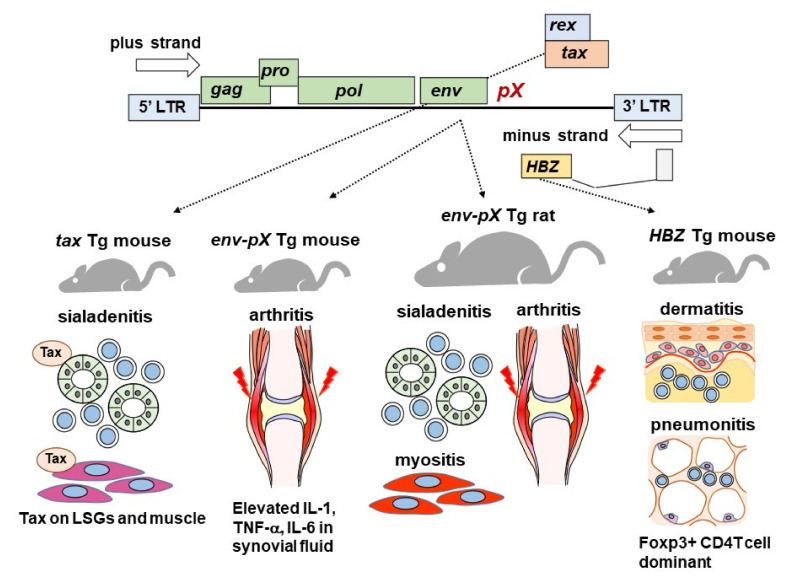
Development of HTLV-1 gene transgenic animals. Four types of HTLV-1 Tg rodents have been created to date. Sialadenitis was observed in *tax* Tg mice, and Tax protein expression was confirmed in salivary glands and muscle tissue by immunoblotting. Arthritis similar to rheumatoid arthritis was observed in *env-pX* mice, and an increase in inflammatory cytokines in synovial fluid was observed. In *env-pX* rats, inflammation was observed in salivary glands, muscles, and joint tissues. In *HBZ* Tg mice, Foxp3-positive T cells were increased, and skin and lung inflammation were confirmed. *HBZ*: HTLV-1 bZIP factor, IL: interleukin, Tg: transgenic, TNF: tumor necrosis factor.

Transgenic (Tg) mice have been used to reproduce the pathophysiology of SS due to HTLV-1 infection. Green et al. created mice in which the *tax* gene which is included in *pX* region that regulates HTLV-1 gene expression was introduced [38] (Figure 1).

The mice were characterized by the proliferation of salivary gland epithelial cells (SGECs) without lymphocyte infiltration in the submandibular, parotid, and labial salivary glands at 4–5 weeks of age. After the proliferation of SGECs, lymphocytic infiltration started around the SGECs, and at 6–8 weeks, lymphocytic infiltration was enhanced, and epithelial islands were formed. The amount of Tax protein in the salivary glands shown by Western blotting increased after 5 weeks of age in accord with the severity of salivary gland inflammation. These results not only showed an association between SS and HTLV-1 but also provided important evidence of the process by which lymphocyte infiltration causes the sialadenitis that occurs in SS.

J.E. Green also reported that transgenic tumor cells in HTLV-1 *tax* Tg mice expressed nerve growth factor [39], and other research groups showed that three other lines of *tax* Tg mouse had neurofibromas that had the potential to activate granulocyte-macrophage colony-stimulating factor [40,41], indicating that *tax* transgenic mice have a phenotype that may be different from the usual SS phenotype. Those HTLV-1 *tax* Tg mice exhibited immunoglobulin abnormalities, and cell lines derived from these mice were used to confirm B-cell proliferation and increased IgM secretion [42].

Iwakura et al. developed HTLV-1 *env-pX* region transgenic mice that showed rheumatoid arthritis (RA)-like chronic arthritis [43]. In these mice, synovitis and an increase in the concentrations of interleukin (IL)-6 and tumor necrosis-alpha (TNF-α) in the joint fluid were also observed, which closely resembles the pathological condition of RA. Lymphocyte infiltration was also observed in the lungs and salivary glands, indicating that SS-like pathology was also induced. Iwakura and his colleagues created *tax*-only transgenic mice and reported that the causative gene for arthritis in *env-pX* Tg mice was *tax*, but they did not mention salivary gland inflammation in their report [44]. These results provide significant evidence that the *tax* gene is able to induce autoimmunity. Since the publication of these studies in the 1980s/1990s, there have been no reports describing the association between *tax* Tg mice and SS.

### 2.2. Env-pX Transgenic Rat

An intraperitoneal or intravenous administration of HTLV-1-infected cells to rats results in persistent HTLV-1 infection [45]. Yoshiki et al. developed multiple HTLV-1-infected rats by administering MT-2 cells, which are derived from ATL [46]. Wistar-King-Aptekman-Hokudai (WKAH) rats are an established rat model that closely resembles HAM [47], which involves chronic inflammation of the thoracic spinal cord and presents with spastic paraplegia and pathological features [48,49]. However, these reports include no mention of SS-like salivary gland formation in these rats.

Yamazaki et al. also created rats in which the *env-pX* region was introduced [50]. Since the introduced region contains a long terminal repeat, it was assumed that *pX* protein expression would occur due to the action of this promoter. Unlike the WKAH rat mentioned above, this *env-pX* Tg rat model exhibited various organ disorders that occur in autoimmune diseases. The *env-pX* Tg rats were characterized by presenting SS-like sialadenitis, but they also presented RA-like synovitis and necrotizing vasculitis. In addition, since *env-pX* Tg rats show expressions of CD80/86 [51] and intercellular adhesion molecule 1 [52], which are important for CD4-positive T-cell infiltration and T-cell activation as they are considered a model of SS and RA induced by HTLV-1.

### 2.3. HBZ Transgenic Mice

Although *tax* activates nuclear transcription factors such as NF-kB, activator protein 1 (AP-1), and cyclic AMP response element binding protein (CREB) [53,54] and a subsequent activation of c-*fos* [55] or *egr*-1/2 [56,57], *HBZ* suppresses the action of *tax*. With respect to the action of transforming growth factor-beta (TGF-b) via Smad or co-activator p300 [58], *HBZ* acts to activate TGF-b via Smad [59], contrary to the action of *tax*. These effects were clarified by the phenotype analysis of *HBZ* Tg mice performed by Satou et al. [60], who also revealed that *HBZ* has two conflicting functions: a transformation of T cells and the induction of chronic inflammation [61]. *HBZ* Tg mice have been confirmed to have chronic inflammation of the lungs and skin [60], but that report also does not mention SS-like salivary gland inflammation.

However, in *HBZ* Tg mice, *HBZ*-induced Foxp3 was observed to exert a reduced action of the commonly known inhibitory Foxp3. Foxp3 is the center of the master regulator of regulatory T cells (Tregs) [62,63]. It is generally thought that Tregs usually suppresses non-Treg cells that induce inflammation through the decreased expressions of accessory signals due to a high expression of CTLA-4 [64]. This seemingly contradictory action of Foxp3 may be the key to explain the pathophysiology of SS associated with HAM, which will be described later.

## 3. The Epidemiology of HTLV-1 Infection in SS

It is estimated that more than 10 million people are infected with HTLV-1 worldwide, but the distribution is highly biased. South Africa, central Australia, the Caribbean islands, South America, and Japan are known as endemic areas [65,66,67]. It was also reported that the conditions of HTLV-1-infected individuals in endemic areas are associated with RA and lung disease, and we observed that bronchiolitis is more frequent in HTLV-1-positive SS patients in Japan’s Nagasaki Prefecture [68].

There are reports from the endemic areas regarding the association between HTLV-1 infection and SS. Lima et al. investigated the cases of 272 anti-HTLV-1 antibody-positive patients at an HTLV-1 clinic in Brazil, and they reported that 21.7% of the patients had sicca syndrome [69]. Interestingly, the anti-Ro/SS-A and La/SS-B antibodies detected in the SS patients were all negative. Elevated serum TNF-a and interferon-gamma (IFN-g) in this group of patients suggested an association between HTLV-1 infection and the pathophysiology of dryness. In another study, the anti-HTLV-1 antibody positive rate was ~4% in approx. 20,000 donors in an endemic area, but as high as ~23% in SS patients, with the odds ratio of 3.1 [70]. We have also observed over the years that HAM patients were associated with a high rate of SS [71,72]; however, the positive rate of anti-Ro/SS-A antibody in SS complicated HAM was approx. 30%, which was significantly lower than the positive rate among anti-HTLV-1 antibody-negative SS patients.

There are reports suggesting an association between HTLV-1 infection and SS even in non-endemic areas. There is a report from France of 15 cases of anti-HTLV-1 antibody-positive SS patients, and although hypergamma globulinemia was observed, no autoantibodies were detected in these cases [73]. The findings of similar anti-HTLV-1 antibody-positive SS cases with poor immune response in both endemic and non-endemic areas are of interest when considering the relationship between HTLV-1 infection and SS.

## 4. The Expression of HTLV-1 in SS

### 4.1. The Expression of HTLV-1 Gene in Labial Salivary Glands (LSGs)

The question of whether HTLV-1 is expressed in the exocrine glands of SS patients is addressed next. The expression of HTLV-1-related genes in LSGs of individuals with SS was reported mainly in the 1990s (Table 1).

Mariette et al. first reported the expression of the HTLV-1 *tax* gene in anti-HTLV-1 antibody-seronegative SS salivary glands in 1993 [74]. The following year, Sumida et al. reported that the LSGs of four of 14 Japanese patients with SS expressed *tax* [75]. With the use of an in situ polymerase chain reaction (PCR) method for the *pX* region, Ohyama et al. confirmed the expression of the HTLV-1 gene only in the infiltrated mononuclear cells (MNCs) of the salivary glands of anti-HTLV-1 antibody-positive SS patients [76]. They also extracted DNA from the patients’ salivary glands and detected *gag*, *pol*, *env*, and *pX* only in the salivary glands of anti-HTLV-1 antibody-positive SS patients. Interestingly, *pX* gene expression was observed in T-cell lines established from anti-HTLV-1 antibody-positive patients, but not in SGEC lines [76].

Tangy et al. identified *gag*, *pol*, and *env* in the salivary glands of HAM patients, but they also observed that *tax* was expressed in some healthy subjects and patients with dry eye and dry mouth [77]. They also performed in situ hybridization (ISH) using the salivary glands of HAM patients, and they observed *tax* expression not only in infiltrating MNCs but also in SGECs. We performed a nested PCR on the salivary gland tissue of anti-HTLV-1 antibody-negative patients from an endemic area, and the results revealed that the *tax* detection rate was low at 18% [78]. Mariette et al. reported that *tax* was identified by PCR in 30% of salivary glands of SS patients, but also in 28% of individuals with other inflammatory diseases [79]. These findings indicate that *tax* gene is detected infrequently regardless of whether the subjects are in an endemic area. Lee et al. later showed that *tax* was present in only 3.8% and *pX*, *p19*, and *env* were not detected in the peripheral blood mononuclear cells (PBMC) of SS patients [80].

The expression of *tax* has thus been a focus of research, but there has been no study on *HBZ* read from the HTLV-1 gene minus strand. Focusing on both *tax* and *HBZ*, we first examined their expression in HTLV-1-infected cell lines. The infected cell line HCT derived from HAM showed a predominant expression of *tax* [81], but expressions of both *tax* and *HBZ* were also observed, and the expression of *tax* was lower in an ATL-derived cell line than in HCT-5 cells. The results of ISH using the same probe revealed the *HBZ* was dominant in SS with ATL, but *tax* was dominant in SS with HAM, clarify a difference in HTLV-1 gene expression between ATL and HAM. Interestingly, *HBZ* and *tax* gene expressions were clearly observed not only in MNCs but also in ductal epithelium. To the best of our knowledge, there is no other published study of *HBZ* expression in SS, and we plan to investigate this in the future.

**Table 1:** Confirmation of HTLV-1-related genes in LSGs from patients with Sjögren’s syndrome.

Target Gene	SS	Control	Author, Journal, Year	Ref.
*tax*	*tax* gene detected in 2/9 (22%) SS LSGs by ISH or PCR	*tax* gene detected in 0/9 (0%) control LSGs	Mariette, *Arthritis Rheum*, **1993**	[75]
*tax*	*tax* gene only was detected in 4/14 (29%) LSGs by PCR	Not performed	Sumida, *Arthritis Rheum*, **1994**	[76]
*pX*	*gag, pol, env* and *pX* gene were detected by PCR and *pX* by in situ PCR hybridization of 5 HTLV-1+SS patients	Not performed	Ohyama, *Arthritis Rheum*, **1998**	[77]
*tax*	*tax* gene was detected in 3/6 (50%) of SS patients from endemic area	*tax* gene was detected in 2/10 (20%) of dryness syndrome from non-endemic area	Tangy, *J Infect Dis*, **1999**	[78]
*tax*	Only 3/17 (18%) of HTLV-1-seronegative SS patients had *tax* gene detected by nested PCR	Not performed	Mizokami, *Scand J Rheumatol*, **1998**	[79]
*tax*	*tax* gene was detected in LSG from 30% of SS patients by PCR	*tax* gene was detected in 28% of inflammatory diseases and 4% of normal subjects	Mariette, *Clin Exp Rheumatol*, **2000**	[80]
*tax, HBZ*	*tax* was detected in MNCs and ductal epithelia cells by ISH in 3 HAM-SS patients. *HBZ* was observed in ATL and HTLV-1 carrier with SS	Neither *tax* nor *HBZ* was found in LSGs from 3 non-SS subjects	Nakamura, *Clin Exp Rheumatol*, **2018**	[82]

HAM: HTLV-1-associated myelopathy, ISH: in situ hybridization, LSG: labial salivary gland, PCR: polymerase chain reaction, RA: rheumatoid arthritis, SGEC: salivary gland epithelial cell, SS: Sjögren’s syndrome.

### 4.2. The Expression of HTLV-1 Protein in LSGs

The detection of HTLV-1-related protein expression at the protein level in vivo is technically difficult, and there are few reports of such a detection. The first report was a *tax* Tg mouse established by Green et al., who observed the expression of Tax protein by conducting Western blotting of salivary glands and muscle tissue [38]. Lee et al. examined the reactivity of Tax protein antibody with HTLV-1 p19 to salivary gland tissue from SS patients, and they reported that 42.4% of the samples responded to the antibody [80].

Shattles et al. stained salivary gland tissues with a monoclonal antibody against p19 belonging to HTLV-1 gag and reported that 31% were positive [82]. However, the anti-HTLV-1 antibody in the patient’s serum was negative, suggesting the possible presence of an endogenous retrovirus. Regarding endogenous retroviruses, Yamano et al. confirmed the existence of type A retrovirus particles in three of 10 salivary glands of SS patients in 1997, suggesting the involvement of a retrovirus in SS [83]. Since then, no reports have clearly shown the direct involvement of retrovirus particles.

In addition to investigating the association between *HBZ* and SS by using ISH, we also examined *HBZ*-induced Foxp3 expression due to the limited expression of *HBZ* in the salivary gland MNCs and duct epithelium of HAM-SS patients [81]; we observed that the expression of Foxp3 was infrequent in anti-HTLV-1 antibody-negative SS LSGs, as reported [84]. However, in the LSGs of HAM-SS patients, we observed Foxp3 expression on MNCs to the extent that there was no significant difference from ATL. This may be due to the paradoxical inflammation-inducing mechanism of Foxp3-positive lymphocytes [61]. In fact, the presence of a group of Foxp3-positive CD4 lymphocytes that can induce inflammation in HAM patients has been reported, along with an association with the proviral load [85].

## 5. The Transmission of HTLV-1 to Salivary Gland Epithelial Cells

### 5.1. The Transmission of HTLV-1 between Lymphocytes

**Figure 2:**
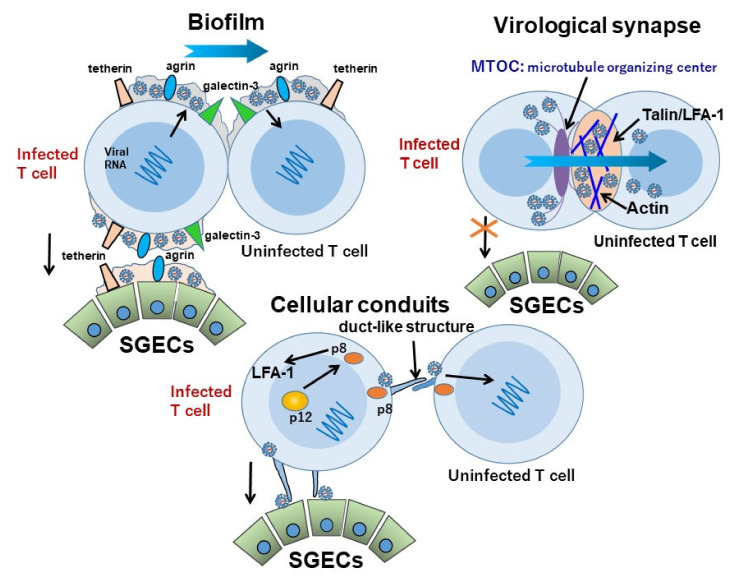
Hypothetical scheme of the transmission of HTLV-1 virions. There are at least three known methods.for transferring viral particles from HTLV-1-infected lymphocytes to non-infected lymphocytes: the biofilm theory, the viral synaptic theory with microtubule organizing center (MTOC) formation, and the cellular conduits theory mediated by the HTLV-1 p8 molecule. It was also suggested that biofilms such as tetherin and agrin are involved in the transfer of virus particles from infected lymphocytes to salivary gland epithelial cells (SGECs). There is also the possibility of transmission via cellular conduits, but it may differ from the transmission between lymphocytes, such as lack of lymphocyte function-associated antigen 1 (LFA-1) accumulation.

Cell-to-cell contact is important for the transfer of viral particles from HTLV-1-infected lymphocytes to non-infected lymphocytes, and there are also reports of the possibility of cell-free infection. The three known types of virus particle migration via cell-to-cell contact are: (1) transmission through viral synapses with microtubule organizing center formation [86,87]; (2) transmission via biofilms such as agrin, tetherin, and galectin on HTLV-1-infected cells to non-infected cells [88,89,90]; and (3) the migration of virus particles via cellular conduits by p8 molecules encoded in HTLV-1 with an accumulation of lymphocyte function-associated antigen 1 (LFA-1) [91,92] (Figure 2). The existence of vasodilator-stimulated phosphoprotein (VASP) that supports the function of HTLV-1 p8 was recently reported [93].

The role of dendritic cells (DCs) in cell-free transmission is gaining attention [94]. Jain et al. investigated the differences in the roles of DC-specific intercellular adhesion molecule-3 (ICAM-3)-grabbing nonintegrin (DC-SIGN) [95] and those of glucose transporter-1 [96] and neuropilin-1 [97,98], which are important for HTLV-1 particle transmission. Their findings demonstrated that DC-SIGN is important for the transmission of HTLV-1 to uninfected T cells.

### 5.2. The Transmission of HTLV-1 between Lymphocytes and SGECs

Only a few studies have investigated the mechanism of infection of HTLV-1 particles to epithelial cells. Martin-Latil et al. evaluated HTLV-1 transmission efficiency by observing passage through the epithelial barrier, and they reported that the epithelial barrier was maintained unbroken during the co-culture of HTLV-1-infected cells and epithelial cells [99]. Other than their study, there has been no report concerning the transmission mode of HTLV-1 before integration into epithelial cells, as shown above.

We reported the involvement of viral synapses, biofilm, and cellular conduits in HCT-5 infected cells from HAM patients [100,101]: the use of viral synapses was not confirmed, but the possibility of their involvement in the other two mechanisms including biofilm and cellular conduits was confirmed (see Figure 2 above). The presence of agrin, tetherin, and galectin on HCT-5 cells was confirmed by immunostaining, but we observed that the HTLV-1 Gag protein was transferred to SGECs together with agrin and tetherin after the co-culture of HCT-5 cells and SGECs. Since the transfer of virus was also confirmed by observations including immuno-electron microscopy, the transfer of virus particles using a biofilm was confirmed. A confirmation of the involvement of cellular conduits was morphologically determined because the accumulation of LFA-1 between lymphocytes was not observed.

### 5.3. The Role of SGECs in HTLV-1 Infection in SS

**Figure 3:**
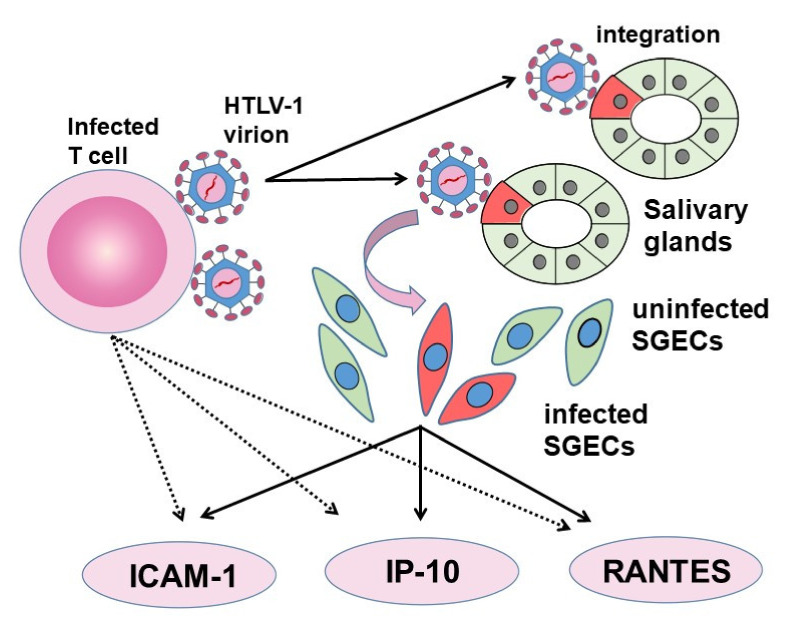
Involvement of HTLV-1 infected SGECs in SS. Virus particles are exposed on the surface of HTLV-1-.infected cell lines, and these infected cell lines produce molecules involved in adhesion, inflammation, and migration, such as ICAM-1, IP-10, and RANTES. HTLV-1 particles migrate to the salivary glands via cell-to-cell contact with infected cells and HTLV-1 DNA becomes integrated into some SGECs. The infected SGECs themselves also have the ability to produce ICAM-1, IP-10, and RANTES, and can be involved in chronic inflammation.

The infection of epithelial cells with HTLV-1 and the question of whether or not immune abnormalities are subsequently induced are important when considering the immune pathology of HTLV-1 infection. Infection of HTLV-1 epithelial cells has been confirmed in thymic epithelial cells [102], retinal pigment epithelial cell lines [103], and synovial cells [104]. We infected synovial cells with HTLV-1-infected cell lines such as HCT-1 and MT-2, and we observed HTLV-1 provirus and the production of granulocyte/macrophage colony-stimulating factor (GM-CSF) from the infected synovial cells [104]. Experiments with primary cultures of SGECs derived from SS patients using HCT-5 confirmed that HTLV-1 can infect SGECs at the protein and gene levels [105] (Figure 3).

We have clarified the time-dependent increases in the levels of intercellular adhesion molecule-1 (ICAM-1) [106], IFN-γ-induced protein 10 (IP-10) [107], regulated on activation, normal T cell expressed and secreted (RANTES) [108], and other molecules involved in inflammation, adhesion, and migration. The expressions of these proteins from SGECs have also clarified the roles of epithelial cells in HTLV-1 infection.

### 5.4. The Effect of HTLV-1 on Autoantibody Production in SS

Secondary lymphoid follicles and the germinal center (GC) play major roles in the production of autoantibodies in autoimmune diseases [109]. Follicular dendritic cells (FDCs) present on the outer layer of the GC are key players [110,111,112] that has the potential to induce autoantibody production from plasma cells after contact with B cells, with the help of follicular helper T cells (Tfh) [113]. We observed that the frequency of GC was lower in anti-HTLV-1 antibody-positive SS LSGs than in anti-HTLV-1 antibody-negative SS [114], and we showed that even in the presence of equivalent inflammation, glandular destruction was less in the anti-HTLV-1 antibody positive patients [115].

**Figure 4:**
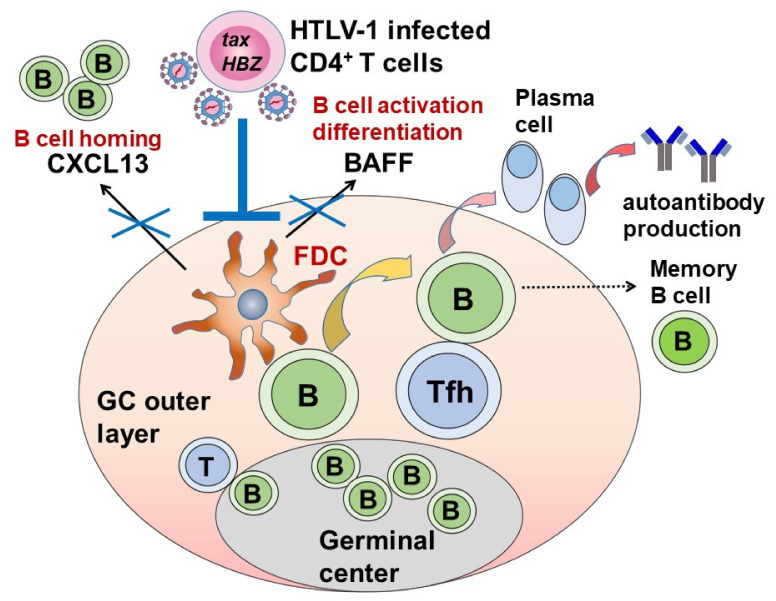
Hypothesis regarding the inhibitory effect of HTLV-1 against autoantibody production system in SS. B cells receive signals from follicular helper T cells (Tfh) after contact with follicular dendritic cells (FDCs) present in the germinal center outer layer. In cooperation with Tfh, memory B-cell production or plasma cell maturation and autoantibody production such as anti-Ro/SS-A antibody occur. Among the autoantibody production pathways, FDC plays a central role in B-cell homing by CXCL13 and B-cell activation and differentiation by BAFF (B-cell activating factor belonging to the tumor necrosis factor family). HTLV-1-infected cells may act directly on FDCs and reduce FDC function by suppressing the production of CXCL13 and BAFF.

To investigate the effect of HTLV-1 on FDCs, we established an FDC-like cell line from human tonsil tissue and observed the effects of HCT-5 and MT-2 cell infections [116]. The co-culture with HTLV-1-infected cell lines resulted in reductions in the concentrations of B-cell activating factor (BAFF) [117,118] and C-X-C motif chemokine ligand 13 (CXCL13) [119], important factors for B cells activation, in the cytoplasm of the FDC-like cell lines (Figure 4), and the reduction of interferon-induced BAFF production.

We also observed that the BAFF and CXCL13 concentrations in the HTLV-1 carrier serum were lower than those in the anti-HTLV-1 negative SS serum [116]. This was the first report showing that HTLV-1 may suppress the effects of FDCs on B-cell activation and autoantibody production.

## 6. Conclusions

The main findings regarding the relationship between HTLV-1 and SS are summarized (Table 2). Tg mice carrying HTLV-1 *tax*, *env-pX*, and *HBZ* showed salivary gland changes similar to those seen in human SS. However, it has yet to be established whether HTLV-1 genes truly cause SS in humans.

In particular, *HBZ* Tg has been confirmed to cause inflammation of the lungs and skin, but it is unclear whether it causes exocrine gland damage similar to SS. In HTLV-1 *tax* and *env-pX* Tg animals, in addition to the lymphocyte infiltration of salivary glands, researchers have also observed plasma cell infiltration and the appearance of rheumatoid factor and anti-DNA antibody in serum. This point contradicts the low frequency of the appearance of autoantibodies such as anti-Ro/SS-A antibody in anti-HTLV-1 antibody-positive SS patients reported in endemic areas such as Brazil and the frequency of autoantibodies in SS with HAM. Whether the appearance of autoantibodies is the main serological abnormality in Tg mice is an issue to be elucidated in light of the low frequency of autoantibodies in SS associated with HAM. We have discovered that the direct effects of HTLV-1 may result in dysfunction of FDCs and a decrease in autoantibody production. However, the mechanisms by which HTLV-1 acts on the autoantibody production system remain a topic for further investigation.

Regarding the expression of the HTLV-1 gene in LSGs, only *tax* is expressed at a low level in non-SS LSGs, but both infiltrating mononuclear cells and SGECs in the LSGs of anti-HTLV-1 antibody-positive SS patients were infected with HTLV-1. In addition, HTLV-1 was integrated after transmission to SGECs via at least biofilm. These observations indicate that HTLV-1 has the ability to infect SGECs and induce inflammation. However, the behavior and effects of HTLV-1 in SS patients remain unclear, and it is necessary to conduct studies using immunological and molecular biological techniques.

In summary, both epidemiological and immunological basic research investigations have begun to reveal the effects of HTLV-1 on SS. However, it cannot yet be ruled out that infiltration of HTLV-1 infected cells and direct infection of SGECs themselves may result in inflammation of the exocrine glands, similar to SS. Further research is essential to confirm whether SS can be recognized as an HTLV-1-related disease.

## Figures and Tables

**Table 2:** Summary of findings suggesting an association between HTLV-1 and Sjögren’s syndrome.

Tg animals with HTLV-1 genes including *tax* and *env-pX* showed sialadenitis that resembled SS. Tax protein was found in *tax* Tg mice.
Epidemiologically, anti-HTLV-1 antibody was highly detected in SS. HAM was frequently complicated with SS, but a low frequency of autoantibodies was observed.
HTLV-1-related genes were detected by PCR or ISH in SS LSGs. A low frequency of *tax* was detectable in LSGs of patients with anti-HTLV-1-seronegative SS or other conditions
HTLV-1 virus particles were transmitted to salivary gland epithelial cells (SGECs) derived from SS at least via biofilm.
HTLV-1 was able to infect SGECs from SS patients, and SGECs after viral integration were able to induce chronic inflammation.
HTLV-1 had an inhibitory function against follicular dendritic cells, and this function is a key component against autoantibody production system.

## Abbreviations

ATL: adult T-cell leukemia, CMV: cytomegalovirus, CXCL: C-X-C motif chemokine ligand, GC: germinal center, HAM: HTLV-1-associated myelopathy, HIV: human immunodeficiency virus, HTLV-1: human T-cell leukemia virus type 1, ICAM: intracellular adhesion molecule, IFN: interferon, IL: interleukin, IP-10: interferon-γ-inducible 10-Kd protein, ISH: in situ hybridization, LFA: lymphocyte function-associated antigen, LSG: labial salivary gland, MNC: mononuclear cell, PCR: polymerase chain reaction, PVL: proviral load, RA: rheumatoid arthritis, RANTES: regulated on activation, normal T cell expressed and secreted, RF: rheumatoid factor, RNA: ribonucleic acid, SGEC: salivary gland epithelial cell, SS: Sjögren’s syndrome, Tg: transgenic, TNF: tumor necrosis factor, WKAH: Wistar-King-Aptekman-Hokudai.
